# Intraprocedural assessment of commissural alignment of the Navitor system using the en-face view

**DOI:** 10.1007/s12928-023-00918-x

**Published:** 2023-02-23

**Authors:** Suguru Hirose, Masaki Nakashima, Yusuke Enta, Kazunori Ishii, Shigeru Toyoda, Norio Tada

**Affiliations:** 1 Department of Cardiology, Sendai Kousei Hospital, 4-15 Hirosemachi Aoba Sendai, Miyagi, 980-0873 Japan; 2 Department of Cardiovascular Medicine, Dokkyo Medical University School of Medicine, 880 Kitakobayashi, Mibu, Tochigi 321-0293 Japan

The positional relationship between the coronary artery and the transcatheter heart valve (THV) commissure is crucial for coronary access following transcatheter aortic valve implantation (TAVI). The ALIGN TAVR study demonstrated optimizing valve alignment using a marker on the delivery system or THV commissural post [1]. However, herein, alignment was assessed using the coplanar long-axis view. The short-axis view could be used to assess accurate commissural evaluation.

The en-face view is useful for comprehending the short-axis orientation in fluoroscopy [2]. Navitor THV (Abbott, CA, USA) has commissure attachment features (CAFs), which provide a marker for the location of each commissure under fluoroscopy. We demonstrated two cases where we used an en-face view to evaluate the commissural alignment of a Navitor THV. Figure 1A–E demonstrates that the commissure was aligned. At 80% deployment of the Navitor 25 mm, aortography was performed at the perpendicular view (Fig. 1A) and en-face view (right anterior oblique [RAO], 42°; cranial [CRA], 25°) (Fig. 1B). Three CAFs were absent from both coronary artery ostia in the en-face view (Fig. 1B–D, ESM Video 1). Computed tomography (CT) images from after TAVI showed that the commissure was in good alignment (Fig. 1E). However, Fig. 1F–J was a misaligned case after Navitor 23 mm implantation. The CAF was in front of both coronary arteries in the en-face view (RAO 52°; CRA 22°) (Fig. 1G–I, ESM Video 2). Because this patient was in her 90 s and her coronary artery was normal, we considered her risk of coronary access would be low and released the THV. Post-CT demonstrated same finding in commissural misalignment and intraoperative findings (Fig. 1J).

**Fig. 1:**
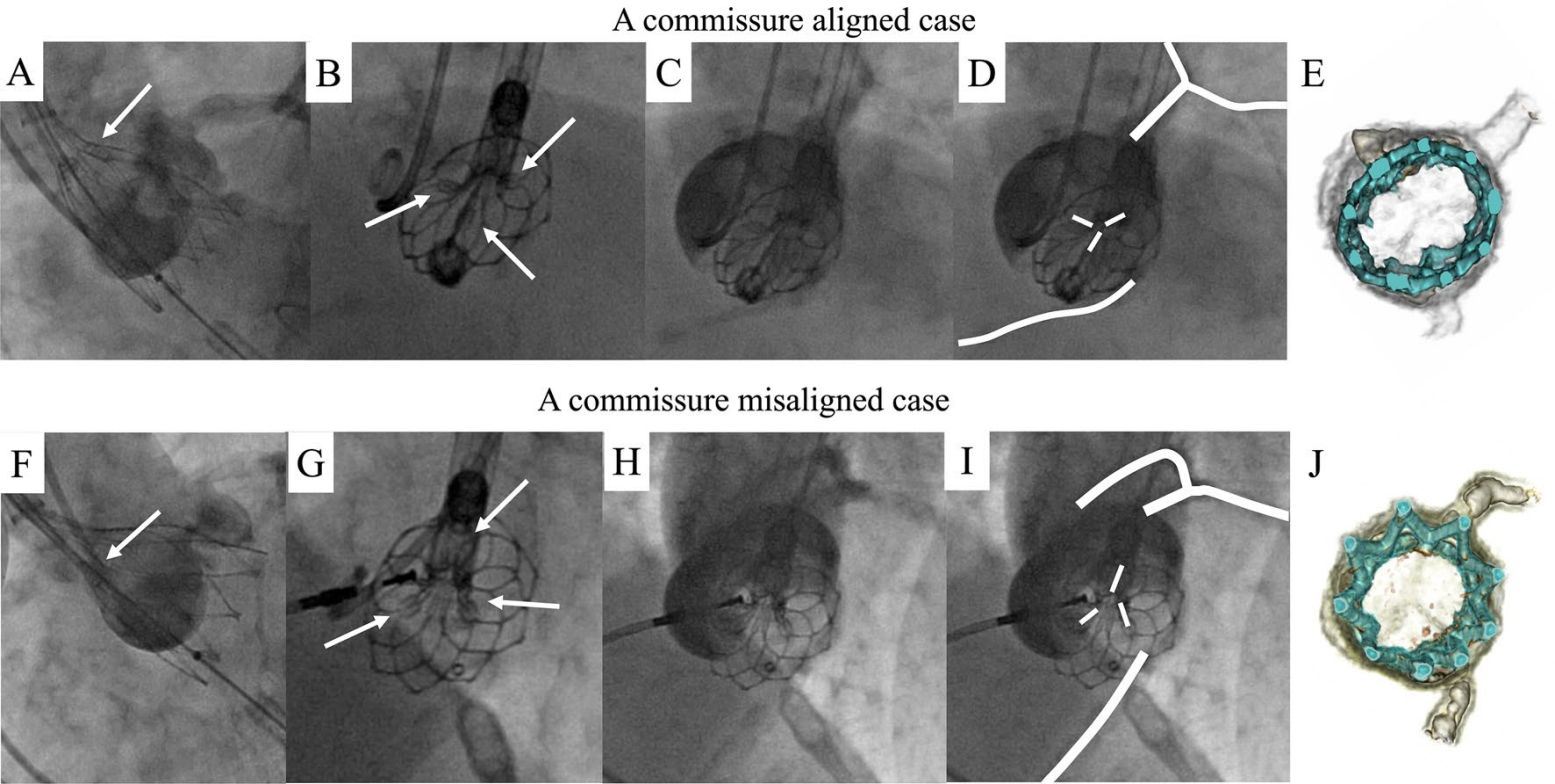
Using the en-face view, implant the Navitor valve. The upper and lower portions represent the aligned and misaligned cases, respectively. **A** Aortography with the Navitor heart valve at 80% development and recognizable CAFs (arrow). **B**–**D** Angiograms taken from the en-face view. White line traces both coronary and CAFs. **E** Commissure alignment was confirmed with the postoperative CT volume rendering (VR) images. **F** Aortography with the perpendicular view in the misalignment case. **G**–**I** CAF overlapped the coronary ostium using the en-face view, resulting in misalignment. **J** Postoperative CT confirmed misalignment. *CT* computed tomography; *CAF* commissure attachment features

This can help recognize short-axis orientation, although it might be challenging to create a true en-face view because the Navitor THV is still connected to the delivery system at 80% deployment. Since Navitor has a recapture function, if misalignment is discovered at 80% deployment in the en-face view, the need for recapture and rotation of the delivery catheter to accomplish commissural alignment can be discussed. In addition, the next-generation Evolut FX system may be also able to do the same assessment because it has fluoroscopic markers that indicate the commissures at the left ventricle edge.

## Supplementary Information

Below is the link to the electronic supplementary material.

Supplementary file1 Aortography with the en-face view in the aligned case. (MP4 312 KB)

Supplementary file2 Aortography with the en-face view in the misaligned case. (AVI 2651 KB)

## Data Availability

Data sharing is not applicable to this article as no datasets were generated or analyzed during the current study.

## References

[1] Tarantini G, Nai Fovino L, Scotti A, Massussi M, Cardaioli F, Giulio R (2022). Coronary access after transcatheter aortic valve replacement with commissural alignment: the ALIGN-ACCESS study. Circ Cardiovasc Interv.

[2] Hirose S, Enta Y, Ishii K, Inoue A, Nakashima M, Nomura T (2022). En face view of the transcatheter heart valve from deep right-anterior-oblique cranial position for coronary access after transcatheter aortic valve implantation: a case series. Eur Heart Case Rep.

